# Electrochemical detection of ascorbic acid in artificial sweat using a flexible alginate/CuO-modified electrode

**DOI:** 10.1007/s00604-020-04510-5

**Published:** 2020-08-27

**Authors:** Bergoi Ibarlucea, Arnau Pérez Roig, Dmitry Belyaev, Larysa Baraban, Gianaurelio Cuniberti

**Affiliations:** 1 Institute for Materials Science and Max Bergmann Center for Biomaterials, Technische Universität Dresden, Dresden, Germany; 2 Center for advancing electronics Dresden (cfaed), Technische Universität Dresden, Dresden, Germany; 3 Present Address: Institute of Radiopharmaceutical Cancer Research, Helmholtz-Zentrum Dresden-Rossendorf e.V., Dresden, Germany

**Keywords:** Ascorbic acid, Nonenzymatic sensor, Electroanalysis, CuO nanoparticles

## Abstract

A flexible sensor is presented for electrochemical detection of ascorbic acid in sweat based on single-step modified gold microelectrodes. The modification consists of electrodeposition of alginate membrane with trapped CuO nanoparticles. The electrodes are fabricated at a thin polyimide support and the soft nature of the membrane can withstand mechanical stress beyond requirements for skin monitoring. After characterization of the membrane via optical and scanning electron microscopy and cyclic voltammetry, the oxidative properties of CuO are exploited toward ascorbic acid for amperometric measurement at micromolar levels in neutral buffer and acidic artificial sweat, at ultralow applied potential (− 5 mV vs. Au pseudo-reference electrode). Alternatively, measurement of the horizontal shift of redox peaks by cyclic voltammetry is also possible. Obtaining a limit of detection of 1.97 μM, sensitivity of 0.103 V log (μM)^−1^ of peak shift, and linear range of 10–150 μM, the effect of possible interfering species present in sweat is minimized, with no observable cross-reaction, thus maintaining a high degree of selectivity despite the absence of enzymes in the fabrication scheme. With a lateral flow approach for sample delivery, repeated measurements show recovery in few seconds, with relative standard deviation of about 20%, which can serve to detect increased loss or absence of vitamin, and yet be improved in future by optimized device designs. This sensor is envisioned as a promising component of wearable devices for e.g. non-invasive monitoring of micronutrient loss through sweat, comprising features of light weight, low cost, and easy fabrication needed for such application.

**Electronic supplementary material:**

The online version of this article (10.1007/s00604-020-04510-5) contains supplementary material, which is available to authorized users.

**Graphical Abstract:**
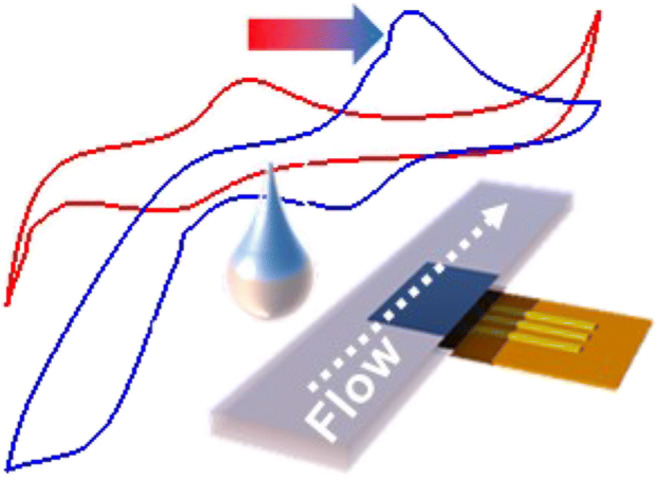
Schematic depiction of the cyclic voltammetry signal change as the sweat flows over the sensor surface.

## Introduction

The long-time known essential ascorbic acid (vitamin C) is a water-soluble molecule that, like other water-soluble vitamins, cannot be stored in the body for a long time [1]; it can be found in secreted body fluids, e.g., sweat [2]. Consequently, heat-exposed activities (e.g., in steel factories) or prolonged sport sessions result in nutrient loss through sweating, including that of ascorbic acid [3], making it necessary to monitor them for the on-time response by dietary intake without delay. Thus, ascorbic acid is an important target for quantitative sensing and monitoring in the biomedical field, relevant also for the food and beverage industry, e.g., for quality control. It is typically measured by high-performance liquid chromatography [4], enzyme-based colorimetric multiwell kits [5], or capillary electrophoresis [6], with limits of detection and linear ranges oscillating between nanomolar and micromolar concentrations. These methods are remote laboratory techniques, with no possibility for on-site measurements.

Taking into account modern day lifestyle and needs, the sensor design principles should evolve correspondingly [7]. In special professional groups like athletes or heat-exposed workers, a miniaturized electrical device with low power requirements and low cost but retaining high sensitivity, integrated in the garment or the skin, would fulfill the requirements of a continuous monitoring for preventive healthcare application. In this context, micro- and nanofabrication techniques play an important role by helping to perform detection processes in a small and low-cost chip [8, 9] with the potential to include flexible characteristics to withstand mechanical stress produced by the movement of the end user [10–13]. Several advanced examples of flexible and even stretchable sensors that could potentially be applied for sweat sensing can be found in the state of the art, based on different materials and focused on different target analytes. The most usual materials that can be found are polymeric, like polydimethylsiloxane, with channels containing dyes for the colorimetric sensing of pH, lactate, glucose, and chloride [14], or polyethylene terephthalate, with electrodes modified with the corresponding enzymes and ionophores for electrochemical and potentiometric detection of glucose, lactate, sodium, and potassium [13]. Other materials can be found as well, like temporary transfer tattoo paper modified with enzyme-modified electrodes for lactate detection [15], or conductive textiles modified with iridium oxide for pH monitoring [16]. Preferably, the fabrication process should be as simple as possible in order to allow a cheap and rapid workflow. Some recent examples of such rapid fabrication include galvanostatic electrodeposition of hydrogels containing enzymes [17], stamping of electrode material with further simple enzyme cross-linking [18], or material vacuum filtration through a mask [19] followed by layer-by-layer or potentiodynamic deposition of receptor layers. Similar concepts would be desired for ascorbic acid measurement.

Most of the recently reported biosensors for ascorbic acid are electrochemical, either enzymatic or nonenzymatic, as it will be described in the following lines. The first group makes use of the natural selectivity of enzymes to discriminate between analyte and possible interferents, being ascorbate oxidase the one used for the present case [20]. This enzyme catalyzes the oxidation of ascorbic acid to dehydroascorbic acid by accepting electrons on its copper center, causing the electron movement that can be measured by the transducer, as it was recently shown with an electrochemical tattoo with an integrated iontophoresis structure to extract sweat [21]. On the other side, nonenzymatic sensors make use of new materials to overcome the limitations of biological receptors in terms of cost and stability [22]. A multitude of attempts have pursued this approach for ascorbic acid determination, for example using carbon-supported PdNi nanoparticles (NPs) [23], carbon nanoplatelets derived from ground cherry husks [24], silver NPs grafted graphene/polyaniline nanocomposites [25], molybdophosphate films [26], graphene oxide/multi-walled carbon nanotubes/gold nanorods combinations [27], ZiNi nanoalloy-modified carbon nanotubes [28], or TiO_2_/reduced graphene oxide nanocomposites [29]. Some sensors provide an added value with the possibility of performing the simultaneous detection of various analytes that typically show overlapping oxidation potentials [30–32]. Krishnan et al. [33] proposed indium tin oxide electrodes with graphitic C_3_N_4_ nanosheets that were modified with nanocomposites made of mesoporous Ag-doped TiO_2_-SnO_2_. Differential pulsed voltammetry experiments allowed to obtain three distinct peaks for ascorbic acid, dopamine, and uric acid in 50-fold diluted urine. The same three analytes can be distinguished as well by using combinations of different materials, among which we may mention: TmPO_4_ and graphene oxide composites [34], assemblies of positively charged Zn-NiAl-layered double hydroxide with negatively charged reduced graphene oxide [35], or three dimensional porous structures of MoS_2_ and reduced graphene oxide composites [36]. Instead, if reduced graphene oxide containing Fe_3_O_4_ nanoparticles is modified with hydroxypropyl-β-cyclodextrin, ascorbic acid, serotonin, and dopamine can be distinguished using the same electrochemical technique [37], as demonstrated in serum samples. On the other hand, it is also known that metal oxide NPs provide a high surface area and good electron transport kinetics [38], which also make them a good candidate for the development of nonenzymatic sensors. Ascorbic acid is known to be a reducing agent for nanostructured copper, being used for NP preparation [39], and as aforementioned, it has a preference for electron donation to the copper center of the ascorbate oxidase enzyme. This property can be exploited for its determination using simpler copper-based nonenzymatic materials, as demonstrated using CuO (copper oxide) nanowires synthesized on Cu foils by a thermal oxidation process at 350 °C for 100 min [40], or with 3D graphene/CuO nanoflowers fabricated by copper electrodeposition on a graphene structure grown by chemical vapor deposition (CVD) on a nickel foam that was later etched [41].

The generality of the aforementioned examples makes use of either high fabrication temperatures, complex equipment (CVD), long fabrication times, or commercially unavailable NPs or nanostructures and nanocomposites that need to be synthesized. In most occasions, the mechanical properties for application in flexible sensors were not tested and in some cases they also result in sensors that measure at a high potential with the resulting increased probability of oxidizing other molecules. Considering the envisioned application of a wearable device for ascorbic acid monitoring in sweat in heat-exposed individuals, minimizing the consumed power (and therefore the working potential) is a must together with good mechanical properties (flexible nature) and ease of fabrication in terms of cost and time. The function in acidic solutions should also be demonstrated.

Here, we report on a new ascorbic acid sensor that combines for the first time the rapid fabrication possibility of the alginate membrane with the encapsulation of the commercially available CuO NPs to exploit their ascorbic acid oxidizing capabilities. The sensor is fabricated via electrodeposition of an alginate membrane using a precursor mixed with CuO NPs on gold electrodes evaporated on a light-weight polyimide support [42]. The alginate membrane, which is reported to be biocompatible and permeable to the diffusion of the electrochemical substrates but impermeable to cells or large particles, has previously been used to trap enzymes for glucose and lactate determination [17, 43] and carbon nanotubes to measure microbial activity [44]. Here, we trap CuO NPs from a commercially available and low-cost nanopowder to make use of their natural interaction with ascorbic acid previously mentioned. The membrane is formed in few minutes in one step and its size can be controlled by the electrodeposition rate [45]. Moreover, it can be easily removed by using a calcium chelating buffer like phosphate-buffered saline (PBS), offering reusability of the electrodes to immobilize a new membrane with possibly different catalysts for additional measurements [17]. The use of simple gold electrodes on polyimide makes it compatible with a variety of techniques like inkjet printing or photolithography and with the future application as a flexible, wearable device. Finally, the determination of ascorbic acid is done at the micromolar range as it is found in sweat [3, 46], and in artificial perspiration solution without interference of any of the other present biomolecules.

## Materials and methods

### Materials and reagents

Support for electrode fabrication: Kapton HN500 (125-μm-thick polyimide film from Dupont, USA, www.dupont.com). Chemicals and reagents: alginic acid sodium salt, calcium carbonate (> 99%), CuO nanopowder (< 50 nm), NaCl (> 99.5%), imidazole (> 99%), KCl (> 99%), and CaCl_2_ (> 98%), NH_4_Cl (> 99.5%), acetic acid (> 99.7%), lactic acid (sodium L-lactate > 99%), D(+)-glucose monohydrate (> 99%), uric acid sodium salt, pyruvic acid sodium salt, L-glutamic acid (> 99%), and urea (> 98%), hexamethyldisilazane (HMDS, > 99%) were purchased from Sigma-Aldrich—Germany (www.sigmaaldrich.com). Filter paper for lateral flow sample delivery: 413-type, VWR, Germany, de.vwr.com/store

### Electrode fabrication and modification

**Fig. 1:**
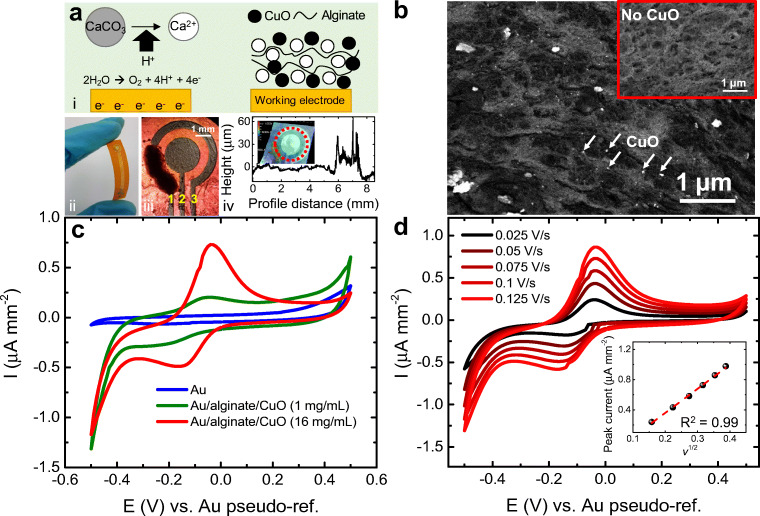
(a) Alginate electrodeposition with CuO nanopowder. (i) Scheme of electrodeposition mechanism. The flexible chip is shown in (ii), with the membrane formed after 140 s electrodeposition time. Yellow numbers indicate electrode function: (1) working, (2) counter, (3) pseudo-reference. Film thickness (10 μm) is shown in (iv), measured along the red dotted circle in inset. (b) SEM imaging of alginate membrane with CuO NPs. Backscattered electrons provide the contrast, with the white spots belonging to the CuO (arrows indicate some examples). Inset shows the membrane without CuO, where no white spots can be seen. (c) Cyclic voltammogram of bare electrodes (blue line) and modified electrodes (red and green), showing the appearance of the redox pair belonging to CuO. (d) Scan rate test and calibration (inset) showing linear relation with square root of the scan rate. All cyclic voltammetries were performed in imidazole buffer at pH 7.4 and room temperature

Gold electrodes were first fabricated on a 125-μm-thick polyimide film (Dupont, USA, www.dupont.com) by a standard photolithography process [11] followed by 100 nm gold thermal evaporation on 3 nm chromium adhesion layer. The chip (Fig. 1a) consisted of three electrodes, using the smallest one (1.2 mm^2^) as working electrode, so that the kinetics at the counter electrode do not limit those at the working one. The area of counter and reference electrodes were 3.8 and 4.1 mm^2^, respectively.

The alginate membrane precursor solution was composed by 1 wt% alginic acid sodium salt and 0.5 wt% calcium carbonate. A total of 16 mg mL^−1^ CuO nanopowder (< 50 nm) was added to the mix as catalyst for the ascorbic acid determination, unless otherwise stated. Twenty microliters of the mix was drop casted on the electrodes (minimum volume to cover reliably the electrodes) and a galvanostatic deposition was carried out using a PalmSens4 potentiostat–galvanostat at a current density of 1.7 A m^−2^ (larger current densities led to electrode delamination) for 140 s (until observing stability of the measured potential). The same system was used for all next cyclic voltammetries and chronoamperometries with DC current. The anodic current produces a water electrolysis generating protons that react with the calcium carbonate, which tends to buffer the solution by consuming the protons and forming Ca^2+^ ions and CO_2_ [45]. The calcium ions interact with the alginate polymers by cross-linking them and creating the membrane, where the CuO NPs get trapped (Fig. 1a-i). The process resulted in a flexible chip with a membrane on the working electrode (Fig. 1a-ii). The drop with the precursor mix could be recovered and used for the deposition on further electrodes. Subsequently, the formed membrane was rinsed in 145 mM NaCl and 10 mM imidazole buffer supplemented with 145 mM KCl to mimic physiological conditions and 10 mM CaCl_2_ to prevent Ca^2+^ ion loss and therefore membrane dissolution. This imidazole buffer, adjusted to pH 7.4, was used for the electrical characterization of the sensor as well as for ascorbic acid measurements in buffer and at room temperature.

### Alginate membrane characterization

The membrane was characterized by optical microscopy, scanning electron microscopy (SEM), and cyclic voltammetry.

Larger current densities than 1.7 A m^−2^ led to electrode delamination, while smaller ones produced weak and unstable membranes that are easily removed. Shorter electrodeposition times (60 s) resulted in soft membranes from which the CuO NPs were easily washed away (Fig. S1, Supplementary Material), while above 140 s the membrane was adsorbed to the electrode in a stable manner during the rinsing steps and the NPs covered the whole working electrode. The evolution of the potential drift due to the galvanostatic deposition process is shown in Fig. S2, where stability starts to be observed after one minute. The resulting membrane after 140 s had a thickness of ca. 10 μm as measured via digital microscopy (Fig. 1a, iv) with a toluidine blue O stained membrane to confer a more homogeneous and non-particulated contrast.

The membranes were prepared for SEM observation following a protocol for hydrogel fixation reported elsewhere [47] but replacing the used buffer for the imidazole-based one to avoid membrane dissolution. First, hydrogels with and without CuO NPs were fixed in 2.5% glutaraldehyde in imidazole buffer for 2 h at room temperature. They were then left at 4 °C for 12 h. After washing twice for 5 min in imidazole buffer, the samples were dehydrated in solutions of increasing ethanol concentration (10, 25, 50, 70, 90, and 100%) for 5 min per concentration. Later, the alginate hydrogel was incubated for 20 min in 50% HMDS diluted in ethanol followed by an additional incubation in a new solution with same characteristics. Finally, the double incubation was repeated in two 100% HMDS solutions. Membranes were left to dry overnight in a desiccator and coated with a 20-nm Au layer by thermal evaporation.

### Bending test

The flexibility of the sensor owing to the mechanical properties of the thin polyimide foil and the soft nature of the alginate was tested by performing cyclic voltammetries before and after controlled bending events. For this, the chip with the electrodeposited membrane was placed on a cylinder of 5-mm radius and bent 1000 times in 10 cycles of 100 bending events each. Cyclic voltammetries were performed in imidazole buffer (pH 7.4, room temperature) between each cycle to observe variations in the signal.

### Ascorbic acid determination

All measurements in buffer were done in the aforementioned imidazole system as non-chelating buffer, adjusted to pH 7.4 and at room temperature. First, cyclic voltammetry at 0.1 V s^−1^ was used to observe the effect on the signal produced by the effect of ascorbic acid. Then, the optimal working potential was chosen for amperometric measurements with different ascorbic acid concentrations at constant pH, to calibrate the sensor and determine the concentration range that could be measured. Next, we proceeded to measure in artificial sweat samples. For this, an artificial perspiration solution was prepared based on the work by Kilic et al. [46], which was composed of 20 g L^−1^ NaCl, 17.5 g L^−1^ NH_4_Cl, 5 g L^−1^ acetic acid, 15 g L^−1^ lactic acid, 0.17 mM glucose, 59 uM uric acid, 0.18 mM pyruvic acid, 0.37 mM glutamic acid, and 10 mM urea. As sweat is a mildly acidic fluid [48, 49], the pH was adjusted to pH 5.5 using HCl. Prior to the measurements directly in this solution, the possible interference by the presence of the organic molecules in the recipe was analyzed. Finally, the artificial perspiration solution was spiked with ascorbic acid and measured in the same way. Considering the difficulties of delivering sweat from skin to a wearable device, we also propose a lateral flow approach as a preliminary design by placing a rectangular filter paper (413-type, VWR, Germany, de.vwr.com/store) in perpendicular to the sensor, and directly drop casting 10 μL samples of the artificial sweat with 100 μM ascorbic acid which were absorbed and redirected toward the sensing area.

## Results and discussion

### Sensor characterization

SEM imaging of backscattered electrons showed the contrast between the membrane material and the trapped CuO (Fig. 1b). The porous membrane appears dark, with white spots belonging to the CuO nanoparticles and aggregates of heterogeneous sizes. In absence of CuO NPs (inset to Fig. 1b), no white spots could be found. An energy-dispersive X-ray spectroscopy found the presence of carbon and oxygen atoms through the whole surface as part of the alginate membrane, as well as calcium cross-linking the alginic acid (Fig. S3). Carbon only lost its presence in the area where the CuO particles agglomerated. On these agglomerates, both copper and oxygen were found. The presence of gold atoms could be seen beneath the membrane, as part of the electrodes. The presence of potassium and silicon atoms can be explained as remaining traces from the prior immersions in the imidazole buffer containing KCl during the rinsing steps, and from the immersion in HMDS in the SEM preparation protocol, respectively.

A cyclic voltammetry of the resulting CuO-containing membrane showed the peaks of the redox pair corresponding to the presence of the CuO NPs (Fig. 1c). The scan rate test between 25 and 125 mV s^−1^ showed a linear increase (*R*^2^ = 0.99, see Fig. S4a for three independent sensors) of the peak amplitude with the square root of the scan rate and with no peak shift (Fig. 1d), indicating a reversible and diffusion controlled system. A slight negative shift was observed only in the cathodic peak; hence, we cannot discard to a certain degree a kinetically controlled behavior [50].

### Bending test

**Fig. 2:**
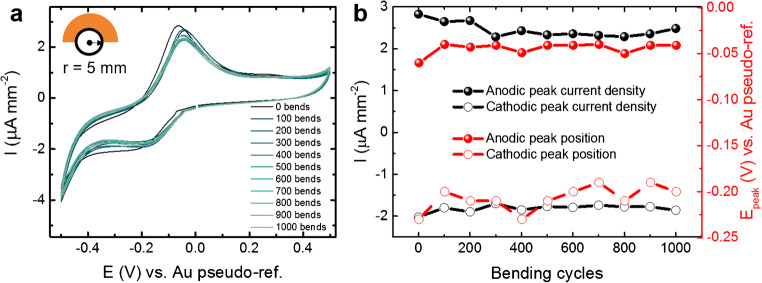
Results of the bending test at 5 mm-radius in imidazole buffer (pH 7.4, room temperature). (a) Cyclic voltammograms and (b) redox peak current density and position before and after each bending cycle. Peak position and current tend to remain around the same values. After an amplitude decrease of less than 1 μA during the first 300 bends, it remained constant for the next 700 events. Inset in (a) shows the principle of the test. The orange film represents the chip on polyimide material, bent onto a 5-mm radius

The measured cyclic voltammograms (Fig. 2a) did not show any significant horizontal shift of the redox peak position, which is the effect that allows measuring ascorbic acid in this work. The amplitude of the anodic peak (Fig. 2b) suffered a slight decrease of 1 μA during the first 300 bending events and remained constant for the next 700 bends. The amplitude decrease for the cathodic peak instead, decreased less than 0.5 μA, showing a high performance even after such high deformation. It must be noted that 5 mm is far beyond the required radius for skin biomonitoring [51].

### Ascorbic acid determination in buffer

**Fig. 3:**
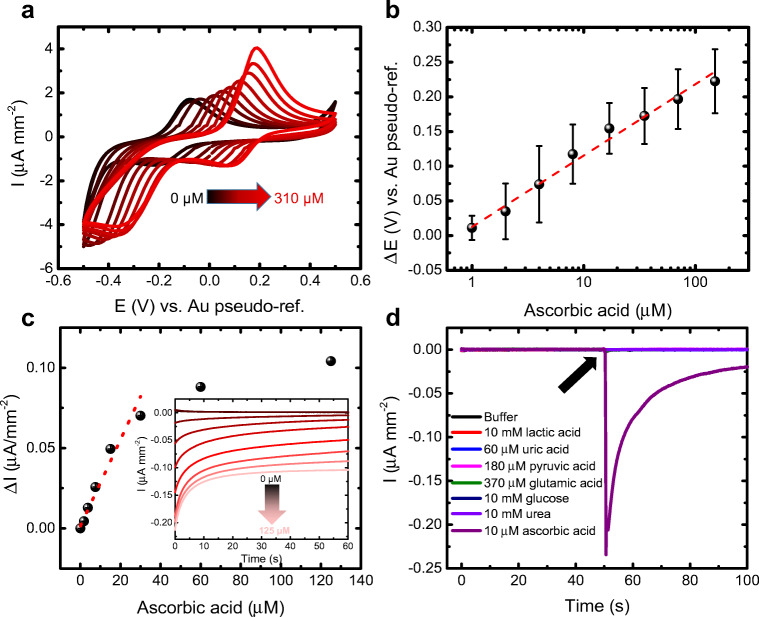
Ascorbic acid determination and analysis of the effect of the interferents in imidazole buffer (pH 7.4, room temperature). (a) Effect of the ascorbic acid on the cyclic voltammogram. The peaks of the CuO redox pairs are shifted toward positive potential. (b) Averaged calibration of the cathodic peak potential shift of 4 independent sensors (error bars as standard deviation of *n* = 4 sensors). (c) Calibration of the amperometry at − 5 mV vs. Au pseudo-ref. electrode in a range between 0 and 125 μM (smallest detected concentration = 1.8 μM). Inset shows the real-time measurements. Further repetitions (*n* = 3) shown in Fig. S5. (d) Interference test showing no response with other than ascorbic acid. The arrow indicates the interferent injection moment

Ascorbate produced a pronounced shift of the redox peaks to a positive potential direction (Fig. 3a), indicating a migration of the CuO through different oxidation states. The linearity with the square root of the scan rate however was not lost (Fig. S4b). Taking the position of the cathodic peak as a reference, the shift increased linearly with the logarithm of the ascorbic acid (Fig. 3b) in a range between 1 and 125 μM, obtaining a limit of detection (LOD) of 1.97 ± 1.03 μM (calculated as the concentration giving a signal 3-fold the standard deviation of the blank) and a sensitivity (*S*) of 0.103 ± 0.004 V log(μM)^−1^ (*y* = (0.32 ± 0.01) + (0103 ± 0.004)*x*; *R*^2^ = 0.99). As seen from the averaged results of four independent sensors, all of them showed the same measurable range.

The fact that the current measured at zero volts in the backwards sweep of the cyclic voltammetry belongs solely to the capacitive current makes it possible to set this potential in a chronoamperometry and measure the shift of the reducing peak as an increasing current as it shifts and approaches the working potential. Therefore, a chronoamperometry was performed at nearly zero volts (− 5 mV vs. Au pseudo-ref. electrode), minimizing the possibility to oxidize other possible interfering biomolecules in a real sample. It is important to note that the measurement is also possible at 0 V; however, − 5 mV was chosen for the sake of stability of the electrical signal. Imidazole buffer containing ascorbic acid in a range between 1.8 and 125 μM was measured. Twenty-microliter samples were drop casted on the sensor and the chronoamperometry was carried out for 60 s for each concentration, observing an increasing negative current together with the increase in analyte concentration (inset to Fig. 3c). The linear range was observed to be up to 30 μM (Fig. 3c, *y* = (0.001 ± 0.002) + (0.0027 ± 2.8 × 10^−4^)*x*; *R*^2^ = 0.95), allowing to perform measurements in the range where ascorbic acid is found in sweat (10–30 μM) [3, 46]. The LOD in this case was 2.3 ± 0.2 μM (calculated using the 3*σ*/*m* criterion where *σ* is the standard deviation of the intercept and *m* is the slope of the calibration plot) and the sensitivity 0.0027 ± 2.8 × 10^−4^ μA μM^−1^ mm^−2^.

A test with three independent sensors using 6 mg mL^−1^ CuO showed, on one hand, that the measurable range was constant and comparable with that obtained using 16 mg mL^−1^ due to the fact that CuO is always in molar excess (Fig. S5). However, further reduction to 1 mg mL^−1^ (voltammogram at Fig. 1c, green line) led to non-reproducible results with highly variable measurable range. We hypothesize that this is caused by the weak redox peaks at low CuO concentration. Therefore, we can conclude that as long as the redox peaks are clearly visible, the CuO concentration is not very relevant, at least starting from 6 mg mL^−1^, concentration that can be chosen as a minimum to measure ascorbic acid reliably, by saving material compared with 16 mg mL^−1^. With measurements of real samples in mind, we performed an interference test by repeating the chronoamperometry in presence of several biomolecules typically found in sweat: lactic acid, uric acid, pyruvic acid, glutamic acid, glucose, and urea. Twenty microliters of buffer was drop casted on the sensor and the measurement was started. After 50 s, additional 20 μL buffer was drop casted with each of the biomolecules, reaching the final concentration found in the artificial perspiration solution to be used later on, including various acids (lactic, uric, pyruvic, and glutamic). The results are shown in Fig. 3d. No response was found for any other than ascorbic acid, being this the smallest concentrated one (10 μM). Neither did the highly concentrated species at 10 mM (lactic acid, glucose, urea). The response for the ascorbic acid was observed instantaneously, showing the fast response of the sensor.

### Ascorbic acid determination in artificial samples

**Fig. 4:**
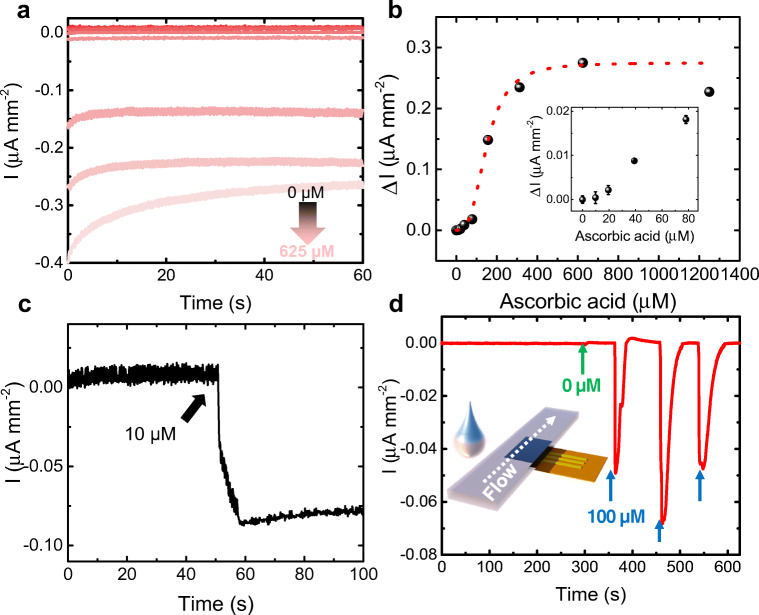
Measurements in artificial perspiration solution, pH 5.5, room temperature. (a) Chronoamperometry and (b) calibration at − 5 mV vs. Au pseudo-ref. electrode. Inset shows magnification of the smallest concentrations. (c) Real-time measurement of 10 μM ascorbic acid (AA) by direct drop casting. (d) Real-time measurement of sweat with and without ascorbic acid by sample delivery through filter paper

Artificial perspiration solutions were prepared with increasing ascorbic acid concentrations and the chronoamperometry was performed at − 5 mV vs. Au pseudo-ref. electrode. Compared with measurements in imidazole, here the measurable range was extended to higher concentrations, with saturation after ca. 300 μM (Fig. 4a, b), which would allow determining the levels found also in blood or a possible excess of ascorbic acid loss through sweat. The current levels were also increased, which is likely coming from the highly complex nature of the solution. The cyclic voltammograms shown in Fig. S6a, SM, support this idea, where the redox peaks and the capacitive current were found to be 4-fold and 2-fold stronger than in imidazole respectively. In spite of that, the levels found in sweat could also be detected, as seen in the real-time measurement of 10 μM ascorbic acid (Fig. 4c). By performing the complete cyclic voltammetry for each ascorbate concentration, the quantification should also be possible by measuring the peak shift (Fig. S6b, SM). Furthermore, one hour incubation in sweat did not produce any degradation of the signal (Fig. S7, SM), demonstrating good stability.

Finally, taking into account the complexity of sweat delivery on a wearable device, we propose the combination of the sensor with a lateral flow approach [52], where a hydrophilic paper allows the gradual passing of a liquid sample. As preliminary test, a rectangular piece of filter paper (413-type filter paper, VWR, Germany, de.vwr.com/store) was directly placed on top of the sensor and 10 μL samples of artificial sweat with 100 μM ascorbic acid was dropped on one side of the paper (inset to Fig. 4d), which absorbed and redirected it toward the sensor, forming clear signal peaks while no signal was observed for the samples without ascorbic acid (Fig. 4d).

Despite the possibility of measuring in the levels and physiological conditions in serum or blood, incubations in fetal bovine serum led to gradual degradation and disappearing of the redox peaks. A previously reported work with CuO entrapped in alginate together with glucose oxidase for glucose sensing however showed successful glucose determination without CuO degradation and ascorbic acid interference, which denotes that the presence of the enzyme might play a role in NP protection. In our case, a direct interaction between CuO and glucose is discarded in the degradation process, as demonstrated in the interference test and the measurements in artificial perspiration solution containing solution, and considering that such interaction requires alkaline conditions [53].

Other nonenzymatic sensors exist with good response at the required micromolar concentrations. A comparison with our work is shown in Table 1. Although many reported works show detection at low potential, ours remains as the one closest to zero volts, and although it was achieved using Au pseudo-ref. electrode, it would definitely help to save battery in a wearable device. The flexible nature of the sensor was also demonstrated and the fabrication was also the simplest, with other sensors requiring long processing times of several hours (> 24 h) with multiple steps, high temperatures (> 350 °C), or high voltage (> 17 V), for nanomaterial synthesis as well as for electrode modification. In addition, our proposed approach performs the measurements in both imidazole (neutral pH) and artificial perspiration solution (acidic), convenient for direct determination in human sweat by consuming low power. Alternatively, measurements by cyclic voltammetry are also possible by analyzing the shift of the redox peaks, in order to choose higher current levels where the noise can be smaller. The main limitations of the proposed system, however, come from the aggregate formation of CuO and the high concentration that is needed despite lower concentrations could already be in molar excess. The concentration is not a major disadvantage considering the low cost of the material, although the reproducibility test shows that 6 mg mL^−1^ is enough and maintains the same measurable concentration range (unlike 1 mg mL^−1^, which leads to unreproducible results). However, the standard deviation in the resulting current levels makes the device more appropriate for semiquantitative analysis, which can still be used to observe increased amounts of vitamin loss, or absence of it. Regarding the issue of aggregate formation, the addition of a surfactant or solvent that provides a better dispersion of the nanopowder in the alginate precursor without affecting the membrane formation could lead to a better homogeneity of the membrane and the obtained results.

**Table 1:** Comparative table of ascorbic acid sensors. Fabrication simplicity is considered based on the number of processing steps, duration, temperature, and applied voltages or currents (− − = very difficult; − = difficult; + = easy; ++ = very easy). *SCE*, saturated calomel electrode

Sensing material	Working potential (V)	LOD (μM)	Sensitivity	Linear range (μM)	Fabrication simplicity
Carbon nanoplatelets derived from ground cherry husks [24]	0.026 vs. Ag/AgCl	1.09	0.20863 μA μM^−1^ cm^−2^	10–3570	+
3-layer sandwich of N-doped graphene, Ag NPs, and polyaniline [25]	1.2 vs. Ag/AgCl	8	28,900–280,500 μM μA^−1^	10–11.460	−
CuO nanowires derived from a self-assembled Cu-Fe nanocube [40]	0.1 vs. Ag/AgCl	0.5	0.07857 μA μM^−1^ cm^−2^	20–400	− −
CuO nanoflowers on 3D graphene foam [41]	0.2 vs. (SCE)	0.43	2.06 μA μM^1^ cm^−2^	20–200	− −
Graphene/copper/phthalocyanine/polyaniline nanocomposites [54]	0.1 vs. Ag/AgCl	0.063	24,460 μA μM^−1^	0.5–120	− −
Redox-active molybdophosphate film [26]	0.4 vs. Ag/AgCl	0.04	0.21021 μA μM^−1^ cm^−2^	1–1500	−
Reduced graphene oxide/Fe_3_O_4_ nanoparticles/hydroxypropyl-β-cyclodextrin nanocomposite [37]	− 0.044 vs. SCE	5	0.01 μA μM^−1^	15–360	+
Graphene oxide/multi-walled carbon nanotubes/Au nanorods [27]	0.036 vs. Ag/AgCl	0.85	7.61 μA μM^−1^ cm^−2^	0.001–0.5 and 1–8000	+
TmPO_4_/graphene oxide composite [34]	− 0.05 vs. SCE	39	0.0013 μA μM^−1^	100–1000	−
Zn-NiAl-layered double hydroxide and reduced graphene oxide superlattices [35]	− 0.1 vs. SCE	0.0135	Not specified	0.5–11	− −
TiO2/rGO nanocomposite-modified glassy carbon electrode [29]	0.22 vs. Ag/AgCl	1.19	1.061 μA μM^−1^ cm^−2^	25–725	−
Pt NP–modified nanoporous AuSn alloy on Ni-buffered flexible carbon fiber paper [31]	0.044 vs. Ag/AgCl	5.51	0.14 μA μM^−1^ cm^−2^	200–2000	− −
Carbon paste electrode modified with CuO-decorated reduced graphene [30]	≈ 0.3 vs. Ag/AgCl	0.009	0.0495 μA μM^−1^	0.04–240	−
ZnNi bimetallic nanoalloy-modified carbon nanotubes [28]	0.19 vs. Ag/AgCl	0.511	Not specified	300–1100	+
Enzyme-modified metalized (1 wt% rhodium) carbon ink [21]	− 0.5 vs. Ag/AgCl	Not specified. Smallest detection: 100	Not specified	100–1000 (amperometry) 100–600 (square wave voltammetry)	+
CuO NPs in alginate (our work)	− 0.005 vs. Au	1.97 (cyclic voltammetry) 2.3 (amperometry)	0.103 V log (μM)^−1^ (cyclic voltammetry) 0.27 μA μM^−1^ cm^−2^ (amperometry)	log1–150 (cyclic voltammetry) 10–30 (amperometry)	++

## Conclusions

The hereby demonstrated sensor provides a very simple solution for ascorbic acid determination in sweat, a very convenient fluid for non-invasive physiological parameter monitoring via skin electronics, by combining for the first time the oxidizing capability of CuO toward the molecule of interest with the facile and quick alginate electrodeposition. Important features of wearable sensors are part of the proposed device, including light weight, determination at ultralow potential vs. Au pseudo-ref. electrode, and compatibility with acidic samples as it is sweat. While both cyclic voltammetry and amperometry are possible, the second approach would allow an easier circuit integration on a wearable device. In addition, the fabrication is done following very few steps and in short time, and commercially available low-cost products are used, which helps to reduce the costs of each final unit. Despite the relatively high concentration of nanoparticles used and the formation of nanoparticle aggregates on the sensing area which could affect reproducibility to some extent, making the system qualitative, future research on the improvement of their dispersion in the precursor solution is a realistic option in order to improve the issue, as aforementioned. Together with the transfer of the technology to electrodes fabricated by screen or inkjet printing, the overall cost could be reduced in the future. Moreover, integration of other catalysts or enzymes for the determination of additional micronutrients, followed by the integration on a wearable electrical device with a miniaturized potentiostat, would enable its implementation in sports or heat-exposed working environments of large duration, protecting the health of the person involved by a timely dietary ascorbic acid intake.

## Electronic supplementary material

ESM 1 (DOCX 2249 kb)
