# Supplementary material for: Electrochemical detection of ascorbic acid in artificial sweat using a flexible alginate/CuO-modified electrode
Source: Mikrochim Acta. 2020 Aug 27;187(9):520. doi: 10.1007/s00604-020-04510-5 (PMC7452922; doi:10.1007/s00604-020-04510-5)
Supplement: Supplementary file 1 — (DOCX 2249 kb) [file 604_2020_4510_MOESM1_ESM.docx]

Electronic Supplementary Material

Electrochemical detection of ascorbic acid in artificial sweat using a flexible alginate/CuO-modified electrode

Bergoi Ibarlucea^1,2^*, Arnau Pérez Roig^1^, Dmitry Belyaev^1^, Larysa Baraban^1,2,3^*, Gianaurelio Cuniberti^1,2^

^1^Institute for Materials Science and Max Bergmann Center for Biomaterials, Technische Universität Dresden, Dresden (Germany)

^2^Center for advancing electronics Dresden (Cfaed), Technische Universität Dresden, Dresden (Germany)

^3^Present address: Institute of Radiopharmaceutical Cancer Research, Helmholtz-Zentrum Dresden-Rossendorf e.V., Dresden, Germany

*Bergoi Ibarlucea: [bergoi.ibarlucea@tu-dresden.de](mailto:bergoi.ibarlucea@tu-dresden.de); Larysa Baraban: [l.baraban@hzdr.de](mailto:l.baraban@hzdr.de)

**Figure S1**. Membrane electrodeposition at (a) 60 s, (b) 140 s, and (c) 210 s.

**Figure S2.** Evolution of the potential during the galvanostatic deposition.

**Figure S3.** Energy-dispersive X-ray spectroscopy of the CuO-doped alginate membrane. (a) Scanning electron microscopy (SEM) of an area showing CuO aggregates trapped in alginate. (b) Spectrum showing the presence of the main elements forming the crosslinked alginate (C, O, Ca), the particle aggregates (Cu and O), and the gold beneath. Insets show their dispersion through the image.

**Figure S4.** Comparison of the scan rate test (a) for three independent sensors and for (b) a sensor before and after drop casting 10 µM ascorbic acid.

**Figure S5**. Ascorbic acid determination with three independent sensors whose membrane was prepared using 6 mg mL^-1^ CuO.

**Figure S6.** Behavior of the alginate membrane with CuO nanoparticles in artificial perspiration solution. (a) Cyclic voltammograms comparing bare electrodes, alginate and CuO modified electrodes in imidazole buffer, and modified electrodes in artificial sweat. (b) Cyclic voltammograms in artificial sweat with increasing ascorbic acid concentration.

**Figure S7.** Stability test of the sensor by performing chronoamperometry in artificial sweat for one hour. No alteration of the signal is observed.
